# Surgical Technique for Varus Deformity Correction of Below-Knee Stump in a Paediatric Patient

**DOI:** 10.7759/cureus.44477

**Published:** 2023-08-31

**Authors:** Nik Alyani Nik Abdul Adel, Ardilla Hanim Abdul Razak, S Suresh Sri Ramulu, Mohd Shukrimi Awang

**Affiliations:** 1 Orthopaedics, Traumatology and Rehabilitation, International Islamic University Malaysia, Kuantan, MYS

**Keywords:** tibiofibular synostosis, corrective osteotomy, varus deformity, paediatric amputation, congenital limb deficiency

## Abstract

Paediatric amputation is one of the treatment options for various indications, namely, trauma, infection, tumour and congenital problems, and some may be born with congenital problems. It differs from adult amputation as they have higher physical demands, and special complications may arise. Stump overgrowth by far is the commonest complication in paediatric transosseous amputation, while varus deformity of the tibia stump was reported sparsely in the literature. The growth discrepancy of the proximal tibia and fibula physis coupled with distal tibiofibular synostosis may have resulted in proximal migration of the fibula, which later resulted in varus deformity of the stump. This will cause difficulty in prosthesis fitting and lead to painful stumps due to the pressure at the abnormal bony prominence. We report a case of congenital limb deficiencies in a 12-year-old male who was treated with below-knee amputation (BKA) and experienced progressive varus deformity of the stump that caused pain during prosthetic wear, which interfered with his gait. He had a varus deformity of 15 degrees of the stump, distal tibiofibular synostosis and proximal migration of the fibula head. As the conservative management by modification of the prosthesis had failed, he underwent open wedge proximal tibia corrective osteotomy, division of the synostosis and reduction of the fibula head. The surgical intervention was successful in alleviating his problem. All efforts must be made to ensure optimum prosthetic fitting in paediatric amputation patients to maintain the patient’s daily lifestyle and activities.

## Introduction

There are various indications for amputation in the paediatric population. Amputation is performed in the management of trauma, infection, tumour or congenital problems. Trauma following a motor vehicle accident or lawn mower injury remains the leading cause of amputation [1,2]. Severe bloodstream infection may also result in amputation, while amputation due to tumours is currently not common [1,2]. Children with congenital limb deficiency can be born with terminal limb deficiency or severe deformities of parts of the long bone, namely, the femur, tibia and/or fibula. These deformities include amniotic band syndrome [2], fibular hemimelia or tibial pseudoarthrosis [1]. These congenital limb deficiencies in some paediatric patients require amputation as a definitive treatment, followed by prosthetic fitting to improve the patient’s quality of life.

Amputation in the paediatric age group is different than in adults as special complications may arise. In addition, children are expected to have higher mechanical and functional demand on the stump and the prosthesis, as their levels of physical activities are different from those of adult amputees [3]. To deal with issues and problems that arise due to amputation in children, it must be balanced with their physical demands. Bone overgrowth in the stump is by far the most common complication of transosseous amputation in children [3]. Other than that, progressive varus deformities of the stump have been reported but very sparsely. This deformity may create problems in prosthetic fitting as it may cause pain or discomfort to the child. Here, we report a case of progressive stump varus deformity following below-knee amputation (BKA) for the treatment of amniotic band syndrome of the leg in a 12-year-old male. The problem was treated with proximal tibial open wedge osteotomy, excision of distal tibiofibular synostosis and reduction of the displaced fibula head.

## Case presentation

**Figure 1:**
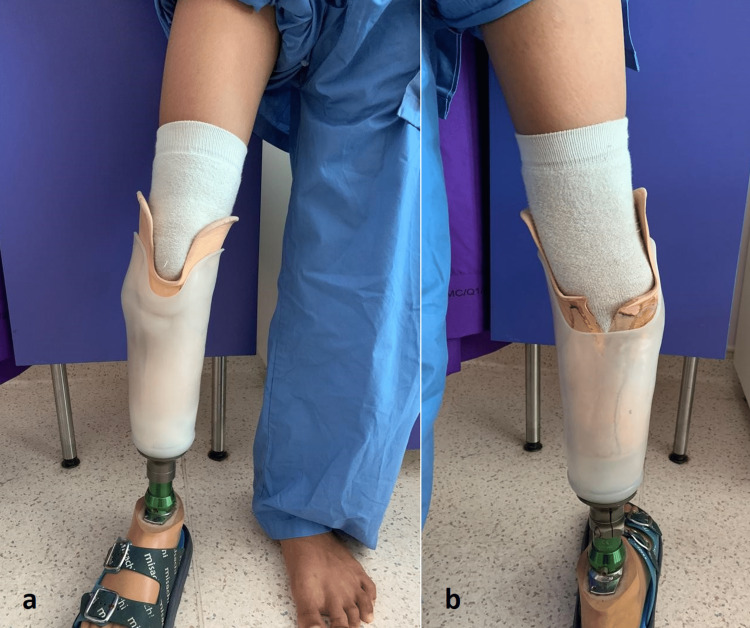
Patient with varus deformity of the stump on modified prosthesis wear in (a) anterior and (b) posterior views.

A 12-year-old male, born with a congenital amniotic band syndrome, was referred to us by the rehabilitation physician due to pain and difficulty in prosthetic fitting. He was initially treated with below-knee amputation and ambulating with a prosthesis. He noticed that his below-knee stump became progressively angulated with bony prominence over the lateral aspect of the knee joint. Prior to the orthopaedic referral, the rehabilitation physician prescribed several prosthetic modifications to accommodate the deformity and reduce abnormal pressure on soft tissue (Figure 1). However, all those measures failed to alleviate the problems.

**Figure 2:**
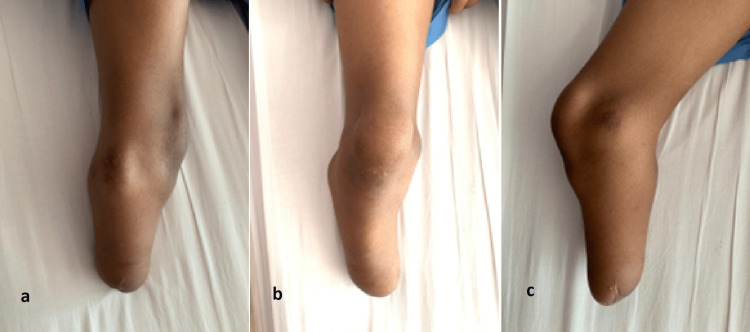
(a) Lateral, (b) anterior and (c) medial views of the stump.

Upon examination of the right lower limb, the below-knee stump appears in varus deformity with the presence of bony prominence over the posterolateral aspect just distal to the knee, which is most likely a fibula head. There were abnormal pressure points evidenced by erythema and mild callosity over the medial femoral condyle, fibula head prominence and distal end of the stump with tenderness (Figure 2).

**Figure 3:**
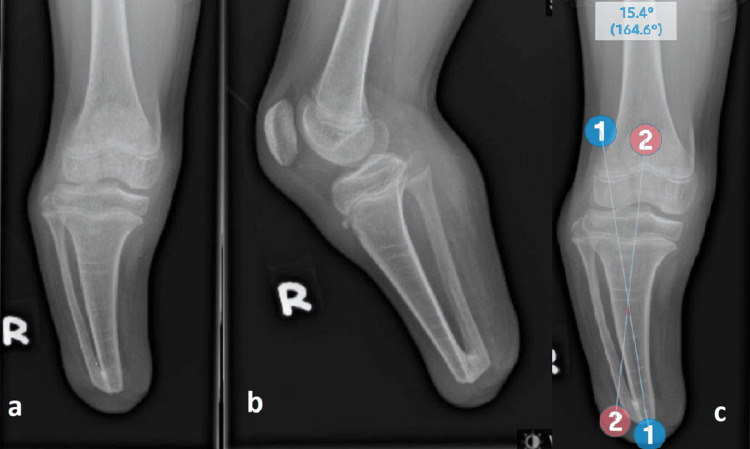
Plain radiograph in (a) anteroposterior and (b) lateral views of the knee joint showing varus deformity of the tibia, proximal migration of the fibula and distal tibiofibular synostosis. (c) Preoperative measurement of the angle.

A plain radiograph of the right knee showed a 15-degree varus deformity with the centre of the rotation axis (CORA) at 3 cm from the distal tibia. There was also the presence of distal tibiofibular synostosis with superoposterior migration of the fibula head. The proximal fibula physis was at the same level as the proximal tibia physis (Figure 3).

**Figure 4:**
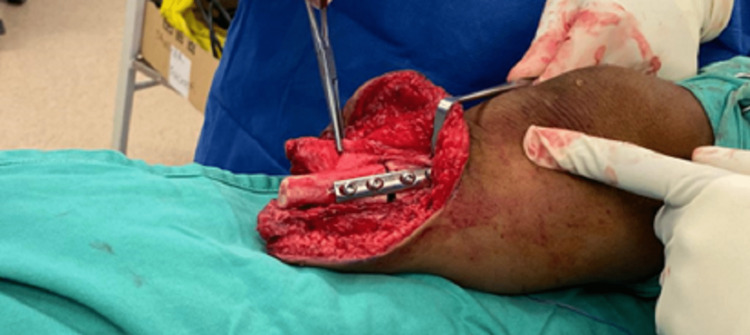
Medial open wedge osteotomy of the tibia after plate osteosynthesis.

As all the conservative management with prosthesis modification of the patient failed, surgical intervention was a necessity at this point. The surgery was performed under general anaesthesia with a pneumatic tourniquet applied to the thigh. An incision was made through a previous distal stump scar, and subperiosteal dissection of the bony portion of the distal tibiofibular synostosis was done. Synostosis division and 2 cm distal fibula shortening were performed. This incision was extended proximally over the medial side to approach the tibia. Open wedge osteotomy was carried out at the CORA. The varus deformity was corrected to around 5-7 degrees valgus using an autologous bone graft from the resected distal fibula. Osteosynthesis was performed using a four-hole small dynamic compression plate (DCP) (Figure 4).

Another incision was made over the lateral aspect of the proximal fibula to reduce the displaced fibula head to its original position, which later was fixed with a 3.5 mm cortical screw to prevent possible future re-displacement. The lateral collateral ligament (LCL) attached to the fibula head was preserved. A fat graft was inserted between the distal tibia and fibula to reduce the risk of reformation of tibiofibular synostosis. The periosteum and soft tissue were repaired with absorbable sutures, and the skin was closed with interrupted sutures using non-absorbable sutures. The knee is kept in extension using the posterior slab. An immediate post-operative plain radiograph showed restoration of tibia alignment with plate osteosynthesis and well-reduced fibula head. The patient was encouraged to ambulate with crutches. The posterior slab was removed two weeks post-operatively.

**Figure 5:**
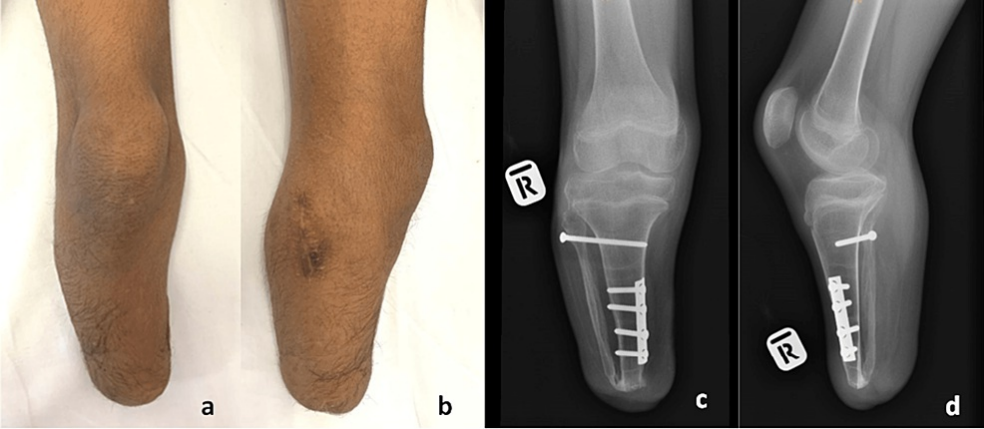
(a,b) Clinical pictures and (c,d) plain radiograph of the patient six months after the surgery. The recurrence of the distal tibiofibular synostosis was observed.

He achieved bone union at 12 weeks post-surgery and was referred back to the rehabilitation physician for prosthetic fitting and gait training. Upon his clinic visit at six months post-operatively, he was able to fit into his prosthesis without any pain or discomfort. Clinical examination revealed normal alignment of the stump and absence of proximal fibula prominence as before (Figure 5). However, the radiograph noted a recurrence of the distal tibiofibular synostosis (Figure 5).

## Discussion

Amniotic constriction band syndrome was first described by Montgomery in 1832 [4] and is also called congenital constriction band syndrome. It is a congenital disorder caused by entrapment of foetal parts in the fibrous band while in utero [5]. Congenital limb deficiencies due to amniotic band syndrome is a sporadic syndrome, and morbidity has been reported ranging from 1:1,200 to 1:15,000 live births [4]. It is diagnosed clinically after birth, but in utero diagnosis can be made through 3D or 4D sonography scan or magnetic resonance imaging (MRI) [5]. The four manifestations of congenital constriction band syndrome are constriction band, lymphedema, acrosyndactyly and, the most severe phenotype, amputation [4,6].

Segal et al. identified 12 transtibial amputation children in their series with progressive varus deformity [7], and Ranade et al. reported that the incidence of angular deformity of the transtibial stump in congenital and acquired causes was 38.1%, including sagittal and frontal plane, or combination [8]. In our case, the patient was delivered with severe deficiency of the leg secondary to amniotic band syndrome. He underwent below-knee amputation and revision of the stump prior to the current presentation of progressive painful varus deformity of the BKA stump and prosthesis fitting problem.

While injury to the physis may be the cause of the angular deformity in traumatic cases [8], distal tibiofibular synostosis was proposed to be the aetiology of angular deformity in non-traumatic cases [7]. Normally, the growth contributed by the proximal fibula physis is more than the proximal tibia physis [7,9]. Hence, the discrepancy of the growth causes more fibula growth than the tibia. The presence of distal tibiofibular synostosis causes the fibula to migrate proximally. In severe cases, it may cause prominence of the fibula head as illustrated in our case.

Non-operative methods such as socket modification and the addition of padding might alleviate the symptoms but may not be well tolerated by some patients. Symptomatic angular deformity is a complex complication that is difficult to treat without surgical intervention. Ten out of 12 patients in the series of Segal et al. and three out of eight patients in the series of Ranade et al. needed surgical intervention due to symptoms of pain and prosthesis fitting problems [7,8]. Angular deformity among these patients was progressive and dynamic in nature in view of growing bone and constant mechanical load on malaligned bone.

The intention for the creation of distal synostosis is usually to prevent complications related to stump overgrowth such as skin perforation, pressure ulcers and difficulties with prosthesis socket fitting. However, in skeletally immature patients, the presence of distal tibiofibular synostosis at the transtibial amputees coupled with relatively greater proximal fibular growth will gradually force the tibia to develop a varus angular deformity [7]. Hence, other alternative methods should be considered, including distal resection, proximal epiphysiodesis, capping of the bony stump using epiphyseal transplants or foreign materials such as silicon, polyethylene or polytetrafluoroethylene (PTFE) felt pads and prolonged skin traction [7,10].

Many surgical techniques have been proposed to treat progressive varus deformity in transtibial amputees, such as proximal tibia corrective osteotomy, lateral hemi-epiphysiodesis and tibial shaft osteotomy. Ranade et al. achieved successful treatment with angular correction using an external fixator [8]. Lineham et al. described the successful correction of an adult transtibial amputation stump who developed malunion of proximal tibia fracture with corrective osteotomy and osteosynthesis with bilateral reconstruction plates [11]. Segal et al. described multiple methods including proximal tibia osteotomy, lateral hemi-epiphyseal stapling or ablation and tibial shaft osteotomy, and all reported good outcomes [7].

We proposed correction with open wedge osteotomy and fixation with small DCP to preserve the length of the stump and prevent an increase in the energy expenditure of the patient with a shorter stump. To resolve the symptom of fibula head prominence, it was reduced to the original level and kept in place with screw fixation to the tibia. Preservation of LCL is important to prevent knee instability when patient weight bear with a prosthesis later. The synostosis was excised, the distal fibula was shortened and the fat graft was inserted between the distal tibiofibular synostosis to reduce the risk of synostosis recurrence. This technique was able to resolve his symptoms and optimize prosthesis fitting. However, since the synostosis recurred in our case, efforts have been taken to regularly monitor for recurrence and ensure optimum prosthetic wear and fitting, but the risk is lesser as the patient grows older.

## Conclusions

In conclusion, progressive varus deformity of the stump may develop in paediatric patients following below-knee amputation due to the formation of distal tibiofibular synostosis and unequal growth over proximal fibula and tibia physis. In severe cases where the prosthesis modification has failed, surgical intervention is needed.

Our technique with corrective open wedge osteotomy of the tibia deformity, shortening and repositioning of the migrated fibula is a valid and reliable option. This technique has proved to immediately alleviate the symptoms and maintain optimized prosthesis fitting.
